# Paraneoplastic pemphigus: initial manifestation of lung cancer

**DOI:** 10.5935/1808-8694.20130045

**Published:** 2015-11-02

**Authors:** Guilherme Webster, Patrícia Maria Sens Marques, Rui Carlos Ortega Filho, Antonini de Oliveira e Sousa, Márcio Cavalcante Salmito

**Affiliations:** a MD - ENT Resident - HSPM-SP; b PhD in Otorhinolaryngology - FCMSCS. Assistant physician - Otorhinolaryngology Department - HSPM-SP; c MD - Otorhinolaryngologist. Public Servant Hospital of São Paulo

**Keywords:** deglutition disorders, dysphonia, pemphigus, skin diseases, vesiculobullous

## INTRODUCTION

The paraneoplastic pemphigus (PNP) is a neoplasia-associated autoimmune disease. It is characterized by a polymorphic mucocutaneous eruption, and its differential diagnoses are: pemphigus vulgaris, multiform erythema, Steven Johnson's syndrome and lichen planus^1^.

It was first described in 1990^2^. PNP patients have reactive antibodies to desmoplakin and desmosomal plaque proteins present throughout their entire epithelium; thus, a more extensive involvement is not surprising^3^.

The goal of the present report is to present a case of paraneoplastic pemphigus, which first manifestation were bullous lesions on the mucosae of the mouth, larynx and nasal septum.

## CASE REPORT

**Figure 1:**
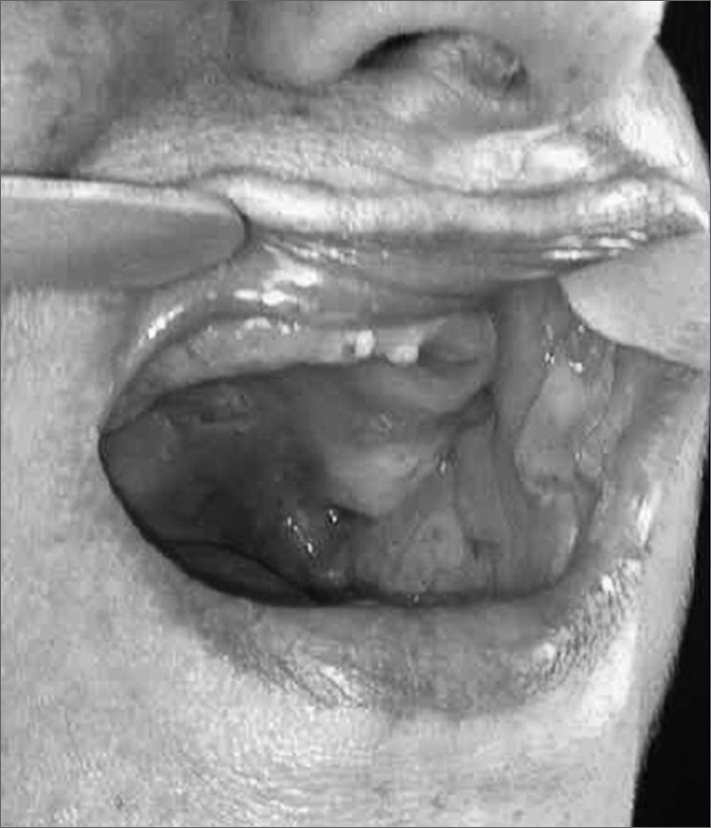
Oroscopy - Bullous and ulcerated lesions on the cheek mucosa and palate.

A 68-year-old woman complaining of epistaxis and painful blisters in her mouth for thirty days, associated with dysphagia and weight loss - which she did not measure. She did not complain of skin lesions. She smoked and had blood hypertension, diabetes and dyslipidemia. Upon clinical exam she had ulcerated and bullous lesions on the palate, cheek mucosa (Figure 1) and lower lip, and also an ulceration on her anterior nasal septum.

She also had ulcerated lesions with fibrin in her larynx and bilateral chorditis, seen upon telelaryngoscopy. Thinking about pemphigus vulgaris, she was started on prednisone, 40 mg/day and we biopsied the lesion on her cheek mucosa - which resulted inconclusive upon light microscopy and immunohistochemistry. After partial improvement only, the prednisone dose was increased to 60 mg/day.

She also developed bullous lesions in her genital and periungual areas, and at this point a new biopsy was carried out and we ordered a test for neoplasia, considering the possibility of paraneoplastic pemphigus: lung function test with restrictive abnormalities. Chest CT scan with subpleural septal thickening, suspected because of the lingula lymphangitis, lower left lobe, as well as medium lobe and left and mediastinal and supraclavicular node enlargements There were also small bullous lesions in the genital and periungual areas when the new biopsy was carried out and we ordered tests for neoplasia, considering the possibility of paraneoplastic pemphigus: pulmonary function test with restrictive abnormalities. Chest CT scan showing subpleural septal thickening - which was suspected because of the lymphangitis on the lingula, lower left lobe, as well as the middle lobe and mediastinum and left supraclavicular nodal enlargements. The digestive track endoscopy showed esophagus acanthosis and enanthematous gastritis.

Maintaining treatment, she enjoyed an important improvement in pain and no new lesions. After two-and-a-half months, she had only a small lesion on the left cheek mucosa. The results from the microscopy and immunohistochemistry suggested pemphigus vulgaris, and the direct immunofluorescence showed mild intercellular and intraepithelial granulous fluorescence, predominating in lower layers of the epithelium. The prednisone dose was then reduce to 40 mg/day. She was worse on the third month of the disease, with enlargement of her neck volume and dyspnea, with new bullous lesions in her cheek mucosa, besides snoring and pulmonary sibilance.

She was admitted to the hospital and a pleural biopsy was carried out, which showed a carcinoma not well-differentiated (not the small-cell type) and, in a later biopsy of a level III neck lymph node on the right side, we defined a moderately differentiated adenocarcinoma (pulmonary metastasis).

She developed respiratory failure and was admitted to the ICU. She died on the fourth month of the disease.

## DISCUSSION

Typical PNP clinical findings include un-treatable stomatitis (more typical) and polymorphic skin lesions. Mucosal involvement is almost always present, ocular, oral, pharyngeal, laryngeal and/or vulvar lesions^4^.

PNP histological findings include ke-ratinocyte necrosis, intraepidermal acantholysis and vacuolar dermatitis on the interface. The immunofluorescence shows an IgG and complement deposits on the cell surface and, frequently, granular or linear complement deposits on the dermis-epidermis junction. The indirect immunofluorescence shows circulating antibodies attached to the simple, columnar or transition epithelium, besides a typical pemphigus pattern. These autoantibodies suffer immunoprecipitation in a protein complex. This is the PNP diagnosis gold standard^5^.

Although this case did not have definitive laboratorial confirmation, the immunofluorescence showed a granular, intercellular, intraepithelial deposit, predominating in the lower layers of the epithelium - which is reported in the literature as a PNP characteristic.

## FINAL REMARKS

The clinical presentation of the bullous lesions may be similar. This case was typical of paraneoplastic pemphigus without definitive laboratorial confirmation.

Thus, the importance of the proper diagnosis for the better treatment of this rare entity.
